# Extracellular and intracellular functions of coiled-coil domain containing 3

**DOI:** 10.1093/jmcb/mjad037

**Published:** 2023-06-01

**Authors:** Sara Omari, Hyemin Lee, Jieqiong Wang, Shelya X Zeng, Hua Lu

**Affiliations:** Department of Biochemistry & Molecular Biology, Tulane University School of Medicine, New Orleans, LA 70112, USA; Tulane Cancer Center, Tulane University School of Medicine, New Orleans, LA 70112, USA; Department of Biochemistry & Molecular Biology, Tulane University School of Medicine, New Orleans, LA 70112, USA; Tulane Cancer Center, Tulane University School of Medicine, New Orleans, LA 70112, USA; Department of Biochemistry & Molecular Biology, Tulane University School of Medicine, New Orleans, LA 70112, USA; Tulane Cancer Center, Tulane University School of Medicine, New Orleans, LA 70112, USA; Department of Biochemistry & Molecular Biology, Tulane University School of Medicine, New Orleans, LA 70112, USA; Tulane Cancer Center, Tulane University School of Medicine, New Orleans, LA 70112, USA; Department of Biochemistry & Molecular Biology, Tulane University School of Medicine, New Orleans, LA 70112, USA; Tulane Cancer Center, Tulane University School of Medicine, New Orleans, LA 70112, USA

**Keywords:** CCDC3, p53, MDM2, lipid metabolism, p63, fatty liver (steatosis), tumor suppressor

## Abstract

Coiled-coil domain containing 3 (CCDC3, also called Favine) is a highly conserved protein initially identified as a protein secreted from adipocytes and endothelial cells in the vascular system with endocrine-like functions. Recently, CCDC3 was also found to function as a nuclear tumor suppressor in breast cancers. Although it is still understudied, CCDC3, since its discovery, has been shown to play multiple roles in lipid metabolism, fatty liver, abdominal obesity, anti-inflammation, atherosclerosis, and cancer. This essay is thus composed to offer an overview of these extracellular endocrine-like and intracellular (nuclear) functions of CCDC3. We also discuss the possible underlying cellular and molecular mechanisms of CCDC3, the implications for clinical translation, and the remaining puzzles about this special molecule.

## Introduction

In 2010, coiled-coil domain containing 3 (CCDC3) was identified by Kobayashi et al. (2010) as a highly conserved protein that is secreted from the vascular system and adipose tissues. CCDC3 mRNAs were initially detected by northern blotting in murine aorta and adipose tissues (Kobayashi et al., 2010), but later found to be expressed in other tissues and cells as well (Eberlein et al., 2010; Ugi et al., 2014). Indeed, CCDC3 was detected in the blood of transgenic CCDC3-overexpressing mice (Liao et al., 2017). A recent study detected CCDC3 in the plasma of wild-type mice, ranging from 40 to 80 pg/ml (Kobayashi et al., 2022). Interestingly, CCDC3 mRNA expression was hormonally and nutritionally controlled in adipose tissues, showing higher levels in obese db/db mice and high-fat–high-sucrose diet-fed mice (Kobayashi et al., 2010). This early study suggested that CCDC3 might play a hormone-like role in the regulation of lipid metabolism potentially involving obesity.

**Figure 1:**
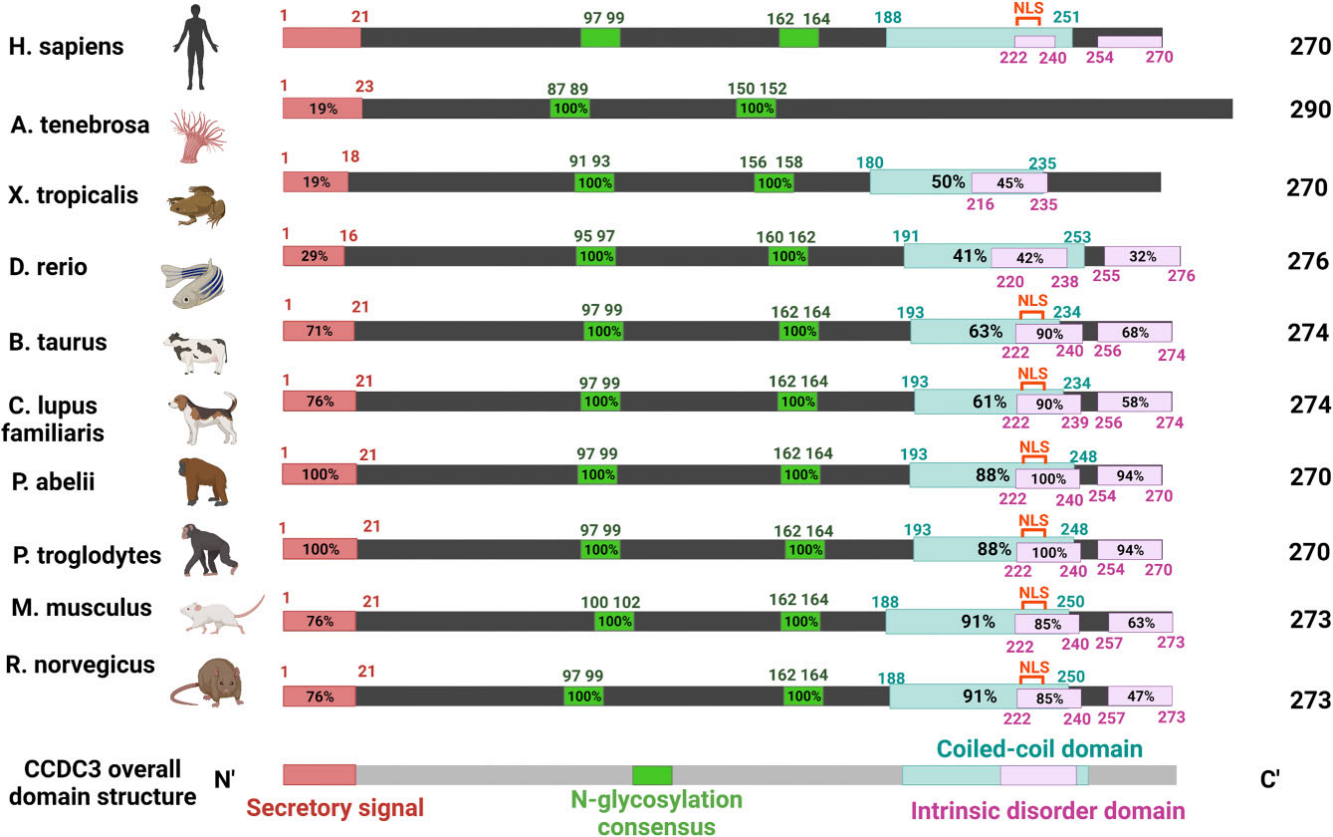
CCDC3 is an evolutionarily conserved molecule. Predicted functional domains of CCDC3 in *Homo sapiens, Actinia tenebrosa, Xenopus tropicalis, Danrio rerio, Bos taurus, Canis lupus familiaris, Pongo abelii, Pan troglodytes, Mus musculus*, and *Rattus norvegicus* are shown. Note that the intrinsic disorder domain is a domain that often maintains equilibrium between states (Babu, 2016). Numbers indicate the positions of amino acids in the polypeptide. The percentage inside the box represents the % homology of this region with the same region in *H. Sapiens*. Sequences were predicted by UniProt, and regions were predicted by UniProt and Eberlein et al. (2010).

Since 2010, more studies have gradually unveiled other functions and regulations of this secretory protein. Surprisingly, although CCDC3 is induced during rat primary adipocyte differentiation (Kobayashi et al., 2010), it is not essential for embryogenesis or postnatal animal development (Kobayashi et al., 2015). Despite this, it is evolutionarily conserved from *Actinia tenebrosa* to *Homo sapiens* (Figure 1; UniProt). It can be secreted by adipocytes via the Golgi pathway (Kobayashi et al., 2010) as a potential endocrine factor that influences the functions of distant organs, such as the liver (Kobayashi et al., 2015; Liao et al., 2017). CCDC3 protein expression is negatively regulated by tumor necrosis factor-α (TNF-α) (Kobayashi et al., 2010; Azad et al., 2014; Liao et al., 2017), isoproterenol (Kobayashi et al., 2010, 2015), and norepinephrine (Kobayashi et al., 2010, 2015) and positively regulated by insulin (Kobayashi et al., 2015) and pioglitazone (Kobayashi et al., 2010), a drug that induces adipocyte differentiation.

On the one hand, we identified the CCDC3 gene as a target of p63 specifically in adipocytes (Liao et al., 2017), both of which are suppressed by TNF-α and upregulated by metformin. Our study suggests a TNF-α–p63–CCDC3 pathway implicated in lipid metabolism regulation in the liver and immune regulation in the blood, which will be further discussed below. On the other hand, CCDC3 may also have intracellular functions, as we recently reported that CCDC3 could be imported into the nucleus of breast epithelial cells where it regulates p53 stability and activity in a positive feedback fashion (Li et al., 2023a). More remarkably, CCDC3 has been linked to human pathological disorders, such as atherosclerosis (Kobayashi et al., 2022), inflammation (Azad et al., 2014), obesity (Ugi et al., 2014), and cancer (Multani et al., 2016; Zhang et al., 2019; Ke et al., 2022; Li et al., 2023a). This essay is thus composed to offer an overview on what we have learned so far about this mysterious molecule, which possesses both extracellular and intracellular functions, though much more about its regulation, biochemical functions, physiological roles, and disease relevance needs to be explored in the future.

## The CCDC3 protein

In humans, the CCDC3 gene is located on chromosome 10 and encodes the CCDC3 protein with 270 amino acids. The protein sequence is evolutionarily conserved among different species (Eberlein et al., 2010; Kobayashi et al., 2010). Important regions of homology between *H. sapiens* and several other species are summarized in Figure 1 using the UniProt alignment tool.

One of the key regions highlighted in Figure 1 is the putative coiled-coil domain in all the listed species except *A. tenebrosa*, in which a low complexity region that often forms helices is predicted by the SMART program (Kumari et al., 2015). As shown in Figure 1, this evolutionarily conserved region in higher eukaryotes is found close to the C-terminus of the amino acid chain. The coiled-coil domain is one of the most ubiquitous functional domains in proteins, with its architecture largely influencing its function (Burkhard et al., 2001). This motif is composed of 2–5 amphipathic α-helices that wrap around each other, forming a supercoil, and has substantial biological utilities in molecular recognition, protein refolding, and structural integrity (Burkhard et al., 2001). It can also mediate oligomerization of subunits in proteins (Burkhard et al., 2001), which may explain why CCDC3 was experimentally confirmed to be a dimer with disulfide bonds between its two subunits (Kobayashi et al., 2010).

CCDC3 is a member of the CCDC family, which is involved in essential biological processes, such as gametogenesis, angiogenesis, and embryonic development (Priyanka and Yenugu, 2021). Polymorphisms in various CCDC family genes have been associated with increased risk of chronic disease and cancer progression (Priyanka and Yenugu, 2021). Hence, CCDC family proteins, such as CCDC3, are studied for their potential implications in tumor suppression or progression.

Another functional domain in the CCDC3 protein is the secretory signal peptide sequence at the N-terminus, which mediates secretion of the protein out of the cell and is cleaved in this process (Kobayashi et al., 2010). The highly conserved nature of this domain, particularly in mammals (Figure 1), suggests that secretory functions of CCDC3 may be retained across several species. Notably, while the CCDC3 protein is secreted from adipose tissues and the vascular system (Kobayashi et al., 2010), it is uncertain exactly where and how CCDC3 is produced in animals. The physiological source and production of CCDC3 warrants further investigation.

Furthermore, N-glycosylation sites are generally predicted using the consensus sequence Asn–Xaa–Ser/Thr (Marshall, 1972). Computer analysis predicted that CCDC3 has two consensus N-glycosylation sites (Kobayashi et al., 2010). Using Cos-7 cells transiently transfected with mouse CCDC3 gene, the protein was experimentally confirmed to be both N-glycosylated and dimeric (Kobayashi et al., 2010). N-glycosylation is implicated in secretory, proliferation, apoptosis, as well as structural organization of the cytoskeleton (Kukuruzinska and Lennon, 1998). This may explain why both consensus regions are highly conserved among species in Figure 1. N-glycosylation patterns have also been correlated with disease progression and drug comparability (Kukuruzinska and Lennon, 1998). This may provide an interesting avenue of research with regard to CCDC3, given its implication in multiple diseases including cancer, which will be discussed later.

The intrinsic disorder domain was predicted in bovine (*Bos taurus*) CCDC3 protein, in which two predicted intrinsic disorder domains extend through the coiled-coil domain into its C-terminus (Eberlein et al., 2010). Intrinsically disordered amino acid regions lack sufficient hydrophobic amino acids required to achieve a three-dimensional, tertiary conformation but have high proportions of polar and charged residues, and thus the resultant proteins often maintain an equilibrium between different structures (Babu, 2016). This feature may afford proteins with the ability to fold in diverse ways, resulting in functional diversity (Dunker et al., 2008). Intrinsic disorder domains are often the sites of post-translational modification (Dunker et al., 2008), increasing functional variety even more.

Interestingly, the first disordered region found in *B. taurus* is almost completely identical amongst the mammals (Figure 1), while the second disordered region shows less inter-species homology. This may be because the first disordered region largely overlaps with the coiled-coil region, which is probably essential to the most important functions of CCDC3. Conservation of the intrinsic disorder domain may specifically provide evolutionary advantages to mammals, since this type of region has vast potential. This may also explain why the intrinsically disordered regions are less conserved in the less complex species (Figure 1).

Recently, we proposed a nuclear localization signal (NLS), LRQARKKGRHL, at CCDC3 C-terminus (Li et al., 2023a). Mutations in this region rendered the protein staying in the cytoplasm rather than in the nucleus (Li et al., 2023a). This signal falls not only in the coiled-coil domain but also in the first, highly conserved intrinsic disorder domain. In fact, NLS is predicted in all the listed mammalian species (Figure 1), indicating a universal importance of the evolutionarily evolved nuclear functions of CCDC3 for higher eukaryotes, which will be discussed later.

It is also worth noting that two domains in the CCDC3 protein are found in more primitive species. One (DUF881) is conserved in a family of bacterial proteins (pfam05949) (Eberlein et al., 2010), with its function still unknown. The other one at the C-terminus (SMC_prok_A) is homologous to a structural maintenance chromosome (SMC) protein family primarily found in archaea (TIGR02169) (Eberlein et al., 2010). SMC proteins are involved in chromosomal segregation and organization and can be found in prokaryotes, eukaryotes, and bacteria. Thus, at least some parts of the CCDC3 protein are highly primitive. The protein has likely evolved to possess even more diverse functions overtime, which will be discussed in the next section.

## Extracellular functions of CCDC3

### Adipose cells: lipid accumulation and liver metabolism

**Figure 2:**
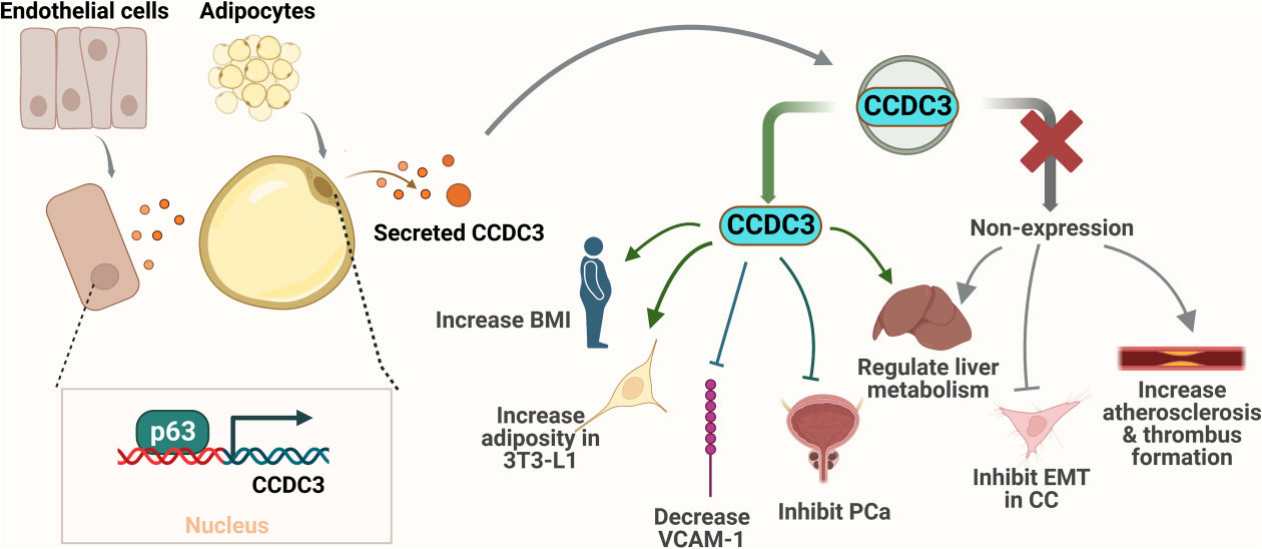
Extracellular functions of CCDC3. Increased CCDC3 is associated with increased omental adipose tissue, BMI, and waist circumference, increased adiposity in 3T3-L1 cells, decreased VCAM-1 production, and decreased migration and invasion of prostate cancer (PCa) cells (Ke et al., 2022). Decreased CCDC3 is associated with decreased migration, proliferation, invasion, and EMT of cervical cancer (CC) cells and increased incidence of atherosclerosis and thrombus formation. CCDC3 regulates hepatic steatosis and lipogenic gene expression in mice and is also a transcriptional target of p63 in adipocytes and possibly ECs. Arrows indicate activation or induction or a flow, while bars indicate suppression.

Since the CCDC3 protein was initially found to be secreted from adipose tissues (Kobayashi et al., 2010; Figure 2), several studies exploring its importance in fat metabolism have ensued. An early study found that cattle with more intramuscular fat had higher CCDC3 mRNA levels in their skeletal muscle (Eberlein et al., 2010). The CCDC3-encoding gene was one of the 23 genes specifically upregulated in human visceral (omental) but not subcutaneous adipose tissues from two abdominally obese males. Eight of these genes, including CCDC3, were shown to have secreted gene products. However, out of the eight secretory genes, only the CCDC3 gene was similarly upregulated in mouse omental adipose tissues from high-fat diet (HFD)-fed mice (Ugi et al., 2014). Furthermore, CCDC3 mRNA expression in human omental (but not subcutaneous) adipose tissues highly correlates with body mass index (BMI) and waist circumference, making CCDC3 a potential biomarker for abdominal obesity (Ugi et al., 2014).

Cell-based studies showed that overexpression of CCDC3 in 3T3-L1 cells leads to increased adipocyte differentiation (Kobayashi et al., 2015). mRNA expression levels of adipogenic genes, including the critical adipogenesis regulator peroxisome proliferator-activated receptor γ (PPARγ), and lipogenic genes, such as fatty acid synthase (FAS) and acetyl-CoA carboxylase α (ACC1), are increased. However, CCDC3 overexpression does not induce PPARγ expression in HEK293T cells, suggesting that CCDC3 lipogenic effect may be specific for adipocytes *in vitro* (Kobayashi et al., 2015).

*In vivo* studies using CCDC3 knockout (KO) mice fed a standard chow diet demonstrated that aged mice exhibit leaner phenotypes compared to control mice, such as having a leaner liver (Kobayashi et al., 2015). Expression levels of lipogenic genes, including FAS, ACC1, and diacylglycerol O-acyltransferase-2 (Dgat2), and carbohydrate-responsive element-binding protein (ChREBP), a regulator of lipogenic enzyme expression, are decreased in adipose tissues of CCDC3 KO mice. Likewise, overexpression of hepatic CCDC3 increases lipogenic gene (FAS and Dgat2) expression levels, although the potential CCDC3 lipogenic effect needs to be validated *in vivo* in non-adipose tissues (Kobayashi et al., 2015). However, expression of the adipogenic gene PPARγ was not affected in adipose tissues of CCDC3 KO mice, suggesting that CCDC3 is predominantly involved in lipogenesis but not adipogenesis *in vivo*.

Interestingly, research in our lab presented somewhat contradictory findings. We found that metformin can activate both TAp63 (a class of p63 protein with a transactivating domain) (Su et al., 2012) and CCDC3 (Liao et al., 2017), while TNF-α can decrease both TAp63 transcriptional activity and CCDC3 protein expression. In addition, the CCDC3 protein has an endocrine-like function and directly targets various mammalian cells, including liver cancer cells (Huh-7, HepG2, and PLC-PRF-5), as shown by immunofluorescent staining (Liao et al., 2017). Lipidomics in Huh-7 hepatic cancer cells treated with CCDC3-containing media presented decreased ceramide and increased long-chain polyunsaturated fatty acid levels, which is a state correlated with fatty liver disease (Luukkonen et al., 2016). Surprisingly, the transgenic (TG) mice ubiquitously overexpressing CCDC3 driven by a cytomegalovirus promoter exhibited relieved glucose intolerance, insulin insensitivity, and hepatic steatosis. The mRNA expression levels of transcription factors involved in lipid accumulation, PPARγ and its target cell death activator-A (CIDEA), were also decreased in CCDC3 TG mice. These results were confirmed by injecting adenovirus expressing ectopic CCDC3 into mice via tail vein (Liao et al., 2017). Finally, HFD mice receiving adenoviral CCDC3-FLAG injection exhibited decreased infiltrating macrophages, liver lipid droplets, and mRNA expression levels of TNF-α and genes involved in *de novo* lipogenesis, suggesting a therapeutic potential of CCDC3 (Liao et al., 2017).

Clearly, the influence of CCDC3 on hepatic metabolism is complex and warrants further research into the implicated mechanisms. On the one hand, CCDC3 deficiency promotes the development of leaner livers in CCDC3 KO mice (Kobayashi et al., 2015). On the other hand, CCDC3 overexpression can decrease adiposity in mouse liver (Liao et al., 2017). The decreased mRNA expression levels of lipogenic genes PPARγ and CIDEA in the liver might account for relieved liver steatosis in HFD-fed CCDC3 TG mice. It is possible that CCDC3 functions via different mechanisms at high vs. low concentrations, leading to different outcomes (Liao et al., 2017).

The CCDC3 protein may directly bind to unidentified receptors on liver cell surfaces or form complexes with ligand receptors via its C-terminal domain (Liao et al., 2017). High-level CCDC3 could inhibit such complex formation, perhaps by sequestering ligands or suppressing endogenous CCDC3 receptors, resulting in similar physiologic outcomes at high vs. low CCDC3 concentrations (Liao et al., 2017). Given that there are two intrinsic disorder domains in CCDC3 coiled-coil region, the protein may exist in equilibrium between several conformational states (Babu, 2016). Studies suggest that a single disordered protein can target multiple ligand partners, and a single ligand partner can be targeted by multiple disordered proteins (Dunker et al., 2008; Oldfield et al., 2008). Abnormally high levels of CCDC3, e.g. in CCDC3-overexpressing cells, may hence change the ligand interaction types for the protein by changing the conformation of its intrinsic disorder domains. Extremely high level of exogenous CCDC3 could also trigger pathways that eventually suppress endogenous CCDC3 function (Liao et al., 2017).

Studies on CCDC3 could lead to novel therapies against liver diseases. For example, nonalcoholic steatohepatitis (NASH) is the term used for an inflamed liver in someone who does not drink excessively (Sheka et al., 2020). The liver may eventually undergo cirrhosis or hepatocellular carcinoma, and it may need to be transplanted (Abdelmalek, 2021). CCDC3 has been associated with abdominal obesity and both upregulation (Kobayashi et al., 2015) and downregulation (Liao et al., 2017) of *de novo* lipogenesis and hepatic steatosis. Thus, a novel therapy for NASH can be developed once the mechanisms by which CCDC3 regulates fatty liver is better understood.

Metformin is a drug for type 2 diabetics to improve their insulin sensitivity by suppressing hepatic gluconeogenesis (An and He, 2016). Since metformin upregulates both p63 and CCDC3 (Liao et al., 2017), we wonder whether CCDC3 plays a role in liver metabolism during this pathological process. Further research may help us determine whether CCDC3 could be used clinically against type 2 diabetes along with metformin.

### Endothelial cells: anti-inflammatory and anti-atherosclerotic

CCDC3 has been shown to be implicated in inflammation and immunity. Both CCDC3 transcript and protein are reduced in the white adipose tissue of oxygen-restricted obese mice, suggesting that CCDC3 could be a marker for hypoxia in the white adipose tissue (Hoevenaars et al., 2019). Endothelial cells (ECs) need to be activated by pro-inflammatory molecules, such as TNF-α, to exert their functions. TNF-α can suppress CCDC3 (Kobayashi et al., 2010), while overexpression of CCDC3 also suppresses TNF-α (Azad et al., 2014), forming a potential negative feedback loop. Specifically, overexpression of CCDC3 in human dermal microvascular endothelial cells (HMECs) decreased both mRNA and protein levels of vascular cell adhesion molecule-1 (VCAM-1), normally induced by TNF-α (Azad et al., 2014). Conversely, depletion of CCDC3 using lentivirus shRNA increased VCAM-1 mRNA and protein levels (Azad et al., 2014).

TNF-α-induced VCAM-1 expression is dependent on NF-κB activation, as treatment with an IKK inhibitor (Bay 11-7082) blocked VCAM-1 expression (Azad et al., 2014). Overexpression of CCDC3 in HMECs also inhibited TNF-α-induced nuclear translocation of p50 and p65 (Azad et al., 2014), which form the dimeric transcription factor NF-κB and function in the nucleus (Bassères and Baldwin, 2006).

Interestingly, VCAM-1 expression levels also dropped in the normal HMECs treated with CCDC3-containing conditioned medium from either HMECs or HEK293T cells transfected with MSCVpac-CCDC3-FLAG (Azad et al., 2014) This suggests that extracellular CCDC3 secreted from HMECs and HEK293 cells can inhibit TNF-α-induced VCAM-1 protein production in HMECs (Figure 2).

TNF-α and NF-κB are both implicated in pro-inflammatory responses (Li et al., 2023b; Galeone et al., 2023; Mussbacher et al., 2023). VCAM-1 expression in ECs is implicated in atherosclerosis (Cybulsky and Gimbrone, 1991; Libby, 2002). Hence, cells neighboring ECs may regulate endothelial inflammation via CCDC3. This raises several enticing questions. Besides HEK293T, whether other non-endothelial cell types expressing CCDC3 can influence endothelial inflammation too? If so, under what conditions? Perhaps multiple cell types and tissues neighboring ECs can help prevent atherosclerosis in a collaborative fashion by enlisting CCDC3 paracrine function. In addition, a negative feedback loop between TNF-α and CCDC3 may contribute to regulating and preventing atherosclerosis (Azad et al., 2014; Figure 2). Further investigating these possibilities would reveal more about how CCDC3 regulates chronic endothelial inflammation and consequent atherosclerosis, which will certainly be conducive to future development of anti-atherosclerotic therapies.

Evidence supporting the anti-atherosclerotic effect of CCDC3 also comes from *in vivo* studies (Kobayashi et al., 2022). ApoE^−/−^Favine^−/−^ double-knockout (DKO) and ApoE^−/−^ (ApoE KO) mice were fed either a Western diet (3–4 months) or normal chow diet (up to 12 months). Aortae from DKO mice fed the Western diet showed more atherosclerotic lesions with bigger lipid cores, more cholesterol crystals, and increased calcified regions, which were independent of common factors causing atherosclerosis. Interestingly, Aortae from ApoE^+/+^Favine^−/−^ mice did not show any signs of atherosclerotic plaques, suggesting that both ApoE and CCDC3 have anti-atherosclerotic effects. Thrombus formation was induced by carotid artery ligation but expression levels of important inflammatory genes were not altered in DKO mice, compared with ApoE KO mice (Kobayashi et al., 2022).

Further analysis of aortae from normal chow diet-fed ApoE KO and DKO mice revealed that actin cytoskeleton signaling and calcium signaling are inhibited and several transcription factors involved in cardiovascular regulation, including myocyte-specific enhancer factor 2c (MEF2C), are decreased in DKO mice relative to ApoE KO mice (Kobayashi et al., 2022). A correlation analysis showed that gene expression changes in unstable regions in human carotid artery plaques and in aortae of DKO mice are positively correlated (Kobayashi et al., 2022). Furthermore, Favine mRNA expression levels are lower in human atheroma plaques than in adjacent regions and decrease as atherosclerosis progresses (Kobayashi et al., 2022).

CCDC3 deficiency decreases mRNA expression levels of MEF2C and its downstream target, KLF2, in human carotid arteries (Kobayashi et al., 2022). MEF2C prevents atherosclerosis formation involving several mechanisms, including the inhibition of the TLR/NF-κB pathway (Xu et al., 2015). Knockdown of CCDC3 using siRNA in human umbilical vein ECs resulted in downregulation of the MEF2C–KLF2–PAI-1/thrombomodulin pathway, suggesting a potential mechanism for the anti-atherosclerotic effect of CCDC3 (Kobayashi et al., 2022). Pursing this line of research further may unveil novel therapies against atherosclerosis by enlisting the anti-inflammatory capacity of CCDC3.

## Intracellular functions of CCDC3

**Figure 3:**
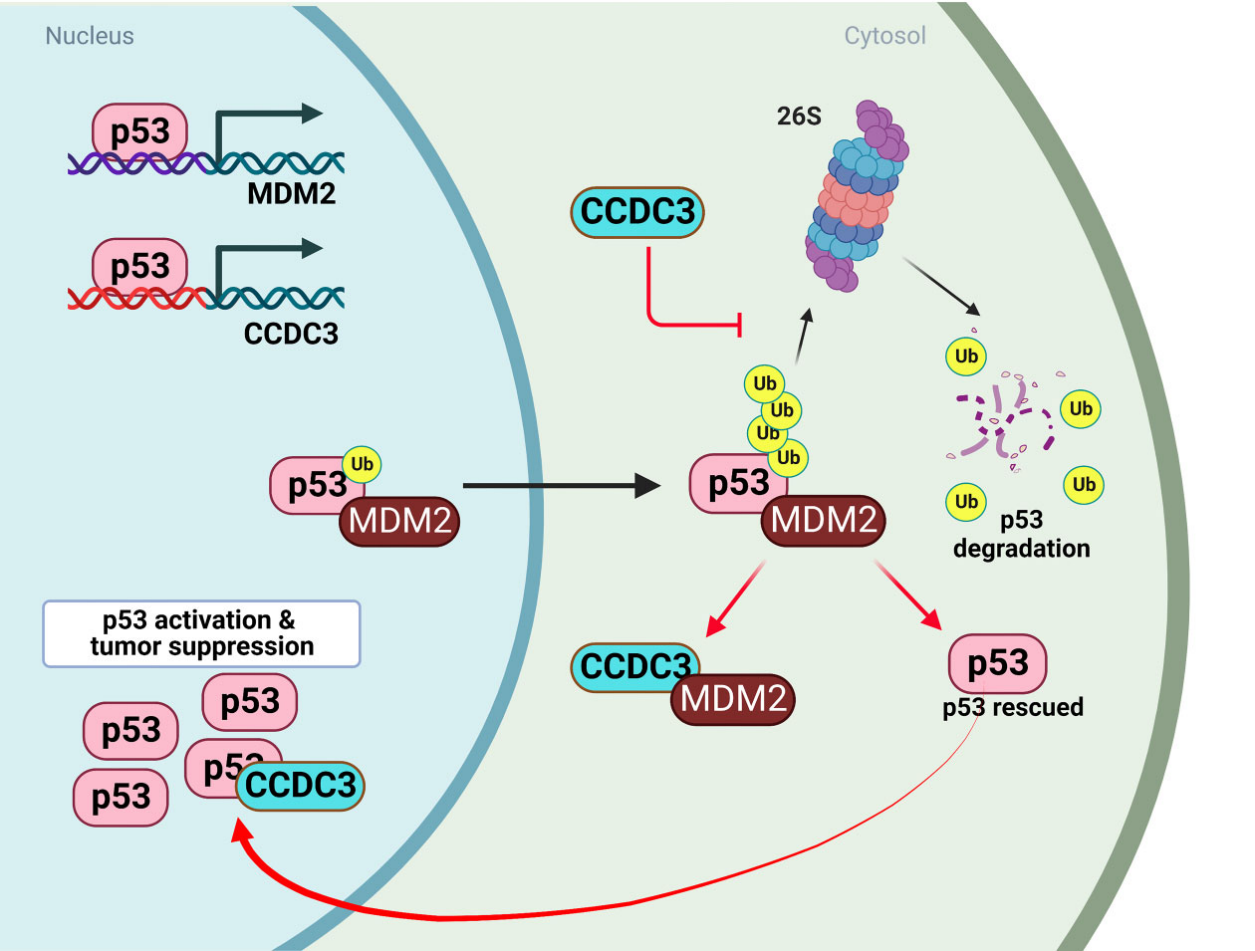
The nuclear function of CCDC3. CCDC3 inhibits MDM2-mediated recruitment of p53 to the 26S proteasome after ubiquitination of p53 by binding to MDM2 and p53 in the cytoplasm. It also binds to p53 in the nucleus to enhance p53 transcriptional activity. CCDC3 is a transcriptional target of p53 in breast epithelial and BrC cells. Arrows indicate activation or induction or a flow, while bars indicate suppression.

Recently, our laboratory discovered novel intracellular/nuclear functions of CCDC3 in breast cancer (BrC), where CCDC3 binds to the C-termini of p53 and MDM2, thereby protecting p53 from degradation by the 26S proteasome, while p53 also upregulates CCDC3 by binding to its promoter region, hence forming a positive feedback loop between p53 and CCDC3 to prevent BrC (Li et al., 2023a; Figure 3).

### CCDC3 and p53 in breast cancer

Through bioinformatics analysis, we found that CCDC3 expression is positively correlated with that of p53 targets (Li et al., 2023a). CCDC3 is highly expressed in breast tissue compared to other tissues, but its mRNA level is markedly reduced in BrC. In BrC patients, a higher level of CCDC3 is well correlated with a higher probability of both survival and survival without relapse. Multiple BrC cell lines expressing wild-type p53 have lower protein and mRNA expression levels of CCDC3 compared with breast epithelial non-cancerous MCF10A cells, confirming that CCDC3 is downregulated in BrC cells and positively correlated with p53 levels in BrC cells (Li et al., 2023a).

Previously, we observed that CCDC3 can bind to the membrane of BrC cells (MCF-7 and MDA-MB-231) (Liao et al., 2017). Recently, we found that CCDC3 can inhibit BrC cell proliferation both *in vitro* and *in vivo* (Li et al., 2023a). However, the binding of CCDC3 to the membrane of MCF-7 and Cal51 cells treated with CCDC3-Myc-His-containing media did not change BrC cell proliferation, and CCDC3 was found mostly in the nucleus of BrC cells (Li et al., 2023a). Since an NLS motif was identified in the CCDC3 C-terminal region (Figure 1), we hence hypothesize that the intracellular but not extracellular CCDC3 affects BrC cell growth and proliferation.

Overexpression of CCDC3 in MCF-7, Cal51, and SKBR7 cells inhibited the proliferation of these BrC cells, whereas knockdown of CCDC3 in Cal51 cells by CRISPR led to the resistance to the anti-cancer drug, 5-FU (Li et al., 2023a). Consistently, when CCDC3-overexpressing Cal51 cells were inoculated into mammary gland fat pads of mice, ectopic CCDC3 suppressed the growth of xenograft tumors via induction of p53 and its target genes, such as p21 (Li et al., 2023a; el-Deiry et al., 1993). Remarkably, CCDC3 can elongate the half-life of p53 from 30 min to 90 min, indicating that CCDC3 upregulates p53 by preventing its degradation. These results demonstrate that CCDC3 can act as a tumor suppressor to inhibit the growth and proliferation of BrC cells and tumorigenesis by activating p53.

Of note, a very recent work reported that CCDC3 knockdown reduces cell viability, inhibits proliferation, and increases apoptosis of MDA-MB-231 cells (Mao et al., 2023). This does not completely contradict to our findings, because MDA-MB-231 cells harbor a hotspot mutant p53, while all the BrC cells used in our study contain wild-type p53 (Li et al., 2023a). Thus, CCDC3 might play different roles in different BrC cells pending on the status of p53, which will be an interesting and important topic for our future exploration.

### CCDC3 prevents MDM2-mediated recruitment of p53 to the proteasome

To elucidate the mechanism by which CCDC3 stabilizes p53, we performed p53 ubiquitination assays (Li et al., 2023a). Surprisingly, CCDC3 enhanced MDM2-mediated ubiquitination of p53. By mutating lysine 63 (K63) and lysine 48 (K48) of the ubiquitin protein, we found that the K48R mutation, but not the K63R mutation, attenuates MDM2-mediated ubiquitination of p53, suggesting that CCDC3-enhanced p53 ubiquitination is K48-dependent. CCDC3 had little effect on p53 ubiquitination in p53 and MDM2 double-null (p53^−/−^/MDM2^−/−^) mouse embryonic fibroblasts, confirming the ubiquitination was MDM2-dependent. Thus, CCDC3 might prevent p53 degradation via a post-ubiquitination mechanism rather than inhibiting its ubiquitination (Li et al., 2023a). GST-fusion protein–protein interaction assays revealed that full-length CCDC3 binds to p53 C-terminus or full-length p53, while MDM2 interacts with CCDC3 C-terminus (Li et al., 2023a). In addition to mediating p53 ubiquitination (Sasaki et al., 2007; Hu et al., 2012), MDM2 can also help recruit ubiquitinated p53 to the 26S proteasome for degradation (Kulikov et al., 2010). By performing co-immunoprecipitation followed by immunoblotting, we found that MDM2 and the S6b subunit of the 19S proteasome, a lid complex of the 26S proteolytic machinery (Goldberg, 2003), are co-pulled down by ectopic CCDC3, while formation of the p53–S6b complex is drastically inhibited by CCDC3 (Li et al., 2023a). Altogether, CCDC3 can prevent the association of p53 with the proteasome by interacting with MDM2 (Figure 3), thus protecting p53 from MDM2-mediated degradation in BrC cells, although more dissections of this process are necessary to further elucidate the exact mechanism.

### CCDC3 is a target of p53

Our previous research suggested that CCDC3 expression can be induced by a p63 isoform TAp63γ, but not by p53, p73, p40, or other isoforms of p63 in H1299 cells and human umbilical vein ECs (Liao et al., 2017). TAp63γ binds to consensus sequence BS1 (−34547, the preferred sequence) and BS2 (−13591), which highly match the preferred consensus sequence of the p53 protein. However, our recent research indicated that p53 can induce the expression of CCDC3 in BrC cells (Li et al., 2023a). First, in MCF-7, Cal51, and SKBR7 cells treated with chemotherapeutic drug (5-FU or doxorubicin), both p53 and CCDC3 protein levels and CCDC3 mRNA level were elevated. Furthermore, knockdown of p53 with shRNA reduced CCDC3 expression, which was reversed by 5-FU or doxorubicin in MCF-7 cells. Also, chromatin immunoprecipitation revealed that p53 binds to two p53-responsive DNA element-harboring regions, BS1 and BS2, at the CCDC3 gene promoter. These results support that the CCDC3 gene is indeed a p53-responsive target gene in breast epithelial and cancerous cells (Figure 3). However, it remains to be investigated why and how p53 possesses tissue- and cell-specific transcriptional target genes (Liao et al., 2017).

Although p53 and p63 belong to the same family of proteins, p63 appears to induce CCDC3 expression in much more organs. Epigenetic factors and tissue differentiation may define this difference. Perhaps only some types of cells, such as breast epithelial and cancerous cells, provide conditions that facilitate the nuclear import of CCDC3, allowing CCDC3 to induce p53 transcription. CCDC3 might also help recruit p53 to its own (CCDC3) promoter region that harbors a p53-responsive DNA element, thus inducing the expression of CCDC3 mRNA and forming a positive feedback loop in some cells but not others. Furthermore, estrogen is known to activate p53 (Dinda et al., 1997). Since MCF-7 cells express estrogen, progesterone, and glucocorticoid receptors (Horwitz et al., 1975), a higher level of p53 may contribute to CCDC3 gene transcription.

Another possibility is that in breast cells, hormones (or other factors) may result in a different conformation of the intrinsic disorder domains at CCDC3 C-terminus, allowing for p53 binding. Furthermore, p53 binds to CCDC3 via its (p53) C-terminal NLS motif (Li et al., 2023a), which is subject to post-translational modification (Harms and Chen, 2006; Ou et al., 2005; Scoumanne et al., 2005), thus causing p53 to act differently from p63.

### Roles of CCDC3 in other cancers

As discussed above, we have uncovered CCDC3 as a tumor suppressor by activating p53 in BrC cells (Figure 3). One remaining question is whether CCDC3 also plays a role in other cancers, either independent or dependent of p53. One study showed that oral cancer risk in tobacco users increases with certain single-nucleotide polymorphisms, including that for CCDC3 (Multani et al., 2016). Another study showed that CCDC3 can inhibit migration and invasion of prostate cancer cells, suggesting that CCDC3 possesses anticancer actions in prostate cancer (Ke et al., 2022), similar to its tumor-suppressive role in BrC (Li et al., 2023a; Figure 3). However, it was also reported that CCDC3 knockdown inhibits migration, proliferation, invasion, and EMT in cervical cancer cell lines (C33 and HeLa) that harbor either mutant p53 or degraded p53 via an E6-AP-mediated mechanism (Zhang et al., 2019), suggesting that CCDC3 might possess a mutant p53-dependent or wild-type p53-independent cancer-promoting action in these cells. Therefore, whether CCDC3 promotes or suppresses cancer growth is likely context-dependent or cell-specific.

**Figure 4:**
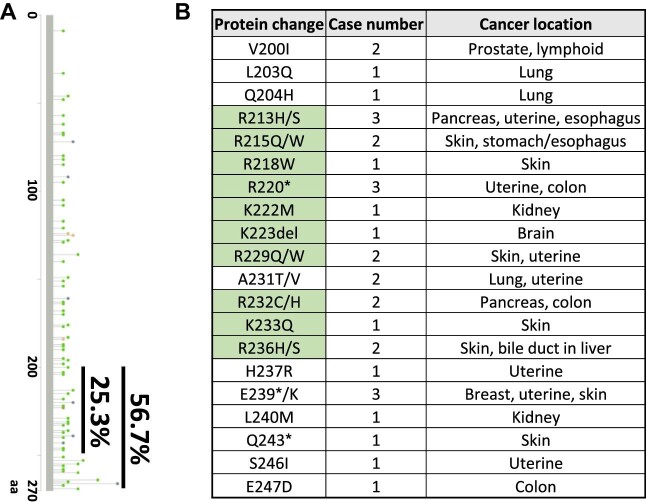
CCDC3 mutations in various carcinomas. (**A**) Schematic graph of CCDC3 mutation sites. A total of 56.7% of mutations occur at CCDC3 C-terminus (200–270 aa), in particular 25.3% in the NLS region (201–250 aa). (**B**) Mutations in CCDC3 NLS region (201–250 aa). The amino acids arginine (R) and lysine (K) are frequently changed (green-highlighted boxes), suggesting that mutated CCDC3 potentially translocates into the cytoplasm and its role is changed (del, in-frame deletion; *, nonsense mutation). Of note, the somatic mutation frequency of the CCDC3 gene is 0.2%. Data were collected by cBioPortal.

Clinically, CCDC3 may be used in anticancer therapies upon further research (Li et al., 2023a). Our analysis of CCDC3 mutations in cancer database revealed that the somatic mutation frequency of CCDC3 is 0.2%, including insertion, deletion, and point mutations (Figure 4). Interestingly, ∼57% of CCDC3 mutations were detected at CCDC3 C-terminus, and 25.3% of the mutations were in the NLS region (Figure 4), supporting that CCDC3 is imported to the nucleus to activate p53 (Figure 3; Li et al., 2023a) and emphasizing the importance of CCDC3 C-terminus in its function as a tumor suppressor. Further studies on these cancer-associated CCDC3 mutations are necessary to unveil the role of CCDC3 in cancer initiation, development, and progression.

## Summary, prospects, and remaining questions

In this essay, we offer an overview of some key findings, though limited, from CCDC3 research since its discovery in 2010, including its roles in abdominal obesity, lipid accumulation, liver metabolism, immunity, and the vascular system as a secretory protein as well as its nuclear function as a tumor suppressor in BrC (Table 1). To execute these biological functions, CCDC3 interplays with several known molecules, including TNF-α, NF-κB, p53, and MDM2, as a downstream transcriptional target of the p53 family (Table 1). Although discussed separately, the extracellular and intracellular functions of CCDC3 cannot be completely dissociated from each other physiologically. It is highly likely that some of the circulating CCDC3 molecules bind to not yet identified membrane-bound receptors and enter cells via endocytosis to function inside the cells as well. It is also likely that CCDC3 molecules bind to not yet identified intracellular receptors or partners to function inside the cells. Apparently, much more studies are required for not only consolidating these reported functions of CCDC3 in animals and humans but also elucidating the underlying mechanisms and unveiling its yet unknown functions. Since CCDC3 works in an endocrine fashion to affect metabolism, synthetic CCDC3 might be used in a similar way as synthetic hormones in hormone replacement therapy. CCDC3 might also serve as a therapeutic agent for human cancers, such as BrC, because it is a short human polypeptide without any apparent toxicity in animals (Liao et al., 2017).

**Table 1:** Possible functions and roles of CCDC3.

Secretory or intracellular	Function	Description
Secretory	Related with abdominal obesity	- CCDC3 level increases in omental adipose tissue (Ugi et al., 2014)- CCDC3 correlates with BMI and abdominal circumference (Ugi et al., 2014)
Secretory	Regulating lipid accumulation	- CCDC3 has a positive correlation with adiposity in 3T3-L1 cells (Kobayashi et al., 2015)- CCDC3 KO mice exhibit leaner phenotypes (Kobayashi et al., 2015)
Secretory	Regulating liver metabolism	- CCDC3 may relieve (Liao et al., 2017) or encourage (Kobayashi et al., 2015) hepatic steatosis in aged mice- CCDC3 may decrease (Liao et al., 2017) or increase (Kobayashi et al., 2015) lipogenic gene expression- CCDC3 is a target of p63 and *vice versa* (Liao et al., 2017)
Secretory	Anti-inflammatory	- CCDC3 prevents endothelial inflammation by inhibiting TNF-α/NF-κB-induced production of VCAM-1 (Azad et al., 2014)
Secretory	Anti-atherosclerotic	- CCDC3 deficiency increases atherosclerosis and thrombus formation in ApoE KO mice (Kobayashi et al., 2022)- CCDC3 deficiency decreases flux through the MEF2C/KLF2 pathway, which normally protects against atherosclerosis (Kobayashi et al., 2022)
Intracellular	Anti- or pro-cancer	- CCDC3 suppresses BrC growth (Li et al., 2023a)- CCDC3 prevents MDM2-mediated recruitment of p53 to the 19S proteasome (Li et al., 2023a)- CCDC3 is upregulated by p53 (Li et al., 2023a)- CCDC3 can inhibit migration and invasion of PC-3 and DU145 prostate cancer cells (Ke et al., 2022)- CCDC3 knockdown leads to the inhibition of migration, proliferation, invasion, and EMT in C33 and HeLa cervical cancer cells (Zhang et al., 2019)

Since the CCDC3 research is at its embryonic stage with limited literature information, there are numerous questions that remain to be addressed, including those discussed above. For example, how CCDC3 targets liver cells. Does it act on hepatocytes via a specific receptor as a secretory protein? If so, what is it or what are they. Does CCDC3 bind to more than one membrane receptor? In addition to p53 and MDM2, what other ‘nuclear receptor’ molecules would CCDC3 bind to? Why does CCDC3 act as a tumor suppressor in some cancers, such as wild-type p53-containing BrC and prostate cancer, but potentially as an oncoprotein in other cancers, such as cervical carcinoma and mutant p53-containing BrC? How exactly does CCDC3 prevent MDM2-mediated recruitment of ubiquitinated p53 into the 26S proteasome? How exactly does CCDC3 regulate immune response and inflammation? Would the cancer-derived CCDC3 C-terminal mutations have biological outcomes, such as promoting tumorigenesis and/or affecting immune response or lipid metabolism? Addressing these outstanding questions would certainly paint a better picture about the biological roles of CCDC3 and the underlying mechanisms, which will be conducive to utilizing this molecule as a potential therapeutic agent against metabolic disorders, immune deficiency, and cancers in the near future.
